# Natural Compound 3β,7β,25-trihydroxycucurbita-5,23(E)-dien-19-al from *Momordica charantia* Acts as PPARγ Ligand

**DOI:** 10.3390/molecules26092682

**Published:** 2021-05-03

**Authors:** Nur Adelina Ahmad Noruddin, Mohamad Faiz Hamzah, Zulfadli Rosman, Nurul Hanim Salin, Alexander Chong Shu-Chien, Tengku Sifzizul Tengku Muhammad

**Affiliations:** 1 National Institutes of Biotechnology Malaysia-Malaysian Institute of Pharmaceuticals and Nutraceuticals (NIBM-IPharm), Ministry of Science, Technology and Innovation, Blok 5A, Halaman Bukit Gambir 11700, Malaysia; nuradelina@nibm.my (N.A.A.N.); faiz@nibm.my (M.F.H.); zulfadli@nibm.my (Z.R.); hanim@nibm.my (N.H.S.); 2 School of Biological Sciences, Universiti Sains Malaysia, Gelugor 11800, Malaysia; alex@usm.my; 3 Centre for Chemical Biology, Universiti Sains Malaysia, Sains@USM, Blok B No. 10, Persiaran Bukit Jambul, Bayan Lepas 11900, Malaysia; 4 Institute of Marine Biotechnology, Universiti Malaysia Terengganu, Kuala Terengganu 21030, Malaysia

**Keywords:** PPAR gamma, type 2 diabetes, natural product, *Momordica charantia*

## Abstract

*Momordica charantia* is a popular vegetable associated with effective complementary and alternative diabetes management in some parts of the world. However, the molecular mechanism is less commonly investigated. In this study, we investigated the association between a major cucurbitane triterpenoid isolated from *M. charantia*, 3β,7β,25-trihydroxycucurbita-5,23(E)-dien-19-al (THCB) and peroxisome proliferator activated receptor gamma (PPARγ) activation and its related activities using cell culture and molecular biology techniques. In this study, we report on both *M. charantia* fruit crude extract and THCB in driving the luciferase activity of Peroxisome Proliferator Response Element, associated with PPARγ activation. Other than that, THCB also induced adipocyte differentiation at far less intensity as compared to the full agonist rosiglitazone. In conjunction, THCB treatment on adipocytes also resulted in upregulation of PPAR gamma target genes expression; AP2, adiponectin, LPL and CD34 at a lower magnitude compared to rosiglitazone’s induction. THCB also induced glucose uptake into muscle cells and the mechanism is via Glut4 translocation to the cell membrane. In conclusion, THCB acts as one of the many components in *M. charantia* to induce hypoglycaemic effect by acting as PPARγ ligand and inducing glucose uptake activity in the muscles by means of Glut4 translocation.

## 1. Introduction

Diabetes mellitus is a type of metabolic disease that is characterised by a high glucose level in blood or hyperglycaemia. This pathophysiological condition occurs when the pancreas does not secrete sufficient insulin or/and the body cannot effectively use insulin leading to hyperglycaemia. This eventually leads to microvascular complications such as diabetic retinopathy, diabetic nephropathy and diabetic neuropathy, as well as macrovascular complications such as coronary artery disease, peripheral arterial disease and stroke [1,2]. A study by the World Health Organization found that the number of adults with diabetes has increased worldwide from 108 million in 1980 to 422 million in 2014 with 1.5 million deaths in 2012 [3]. The number of diabetes cases has increased to 463 million in 2019 [4].

In 2019, World Health Organisation published an update on the classification of diabetes mellitus which comprises of type 1 diabetes, type 2 diabetes (T2DM), hybrid forms of diabetes, other specific types, unclassified diabetes and hyperglycaemia first detected during pregnancy [5]. Of all these types, T2DM is the highest prevalence form affecting 90–95% of diabetic patients [5]. T2DM, which is also known as non-insulin-dependent diabetes, is mainly characterised by inability of peripheral tissues—mainly adipocytes, skeletal muscle and liver—to efficiently utilise insulin. Insulin resistance causes impaired glucose uptake which leads to excessive levels of blood glucose [6,7]. In addition, due to continuous high levels of glucose in blood, β-cells of pancreas exhaustively secrete insulin which may lead to the destruction of the insulin-producing cells [8]. Besides reduced β-cell mass, deficiency in β-cell function is another contributing factor leading to hyperglycaemia [9]. Recently, it has been postulated that fat accumulation in the liver and pancreas are the causes of β-cell dedifferentiation and loss of function, being that, hyperglycaemia in T2DM patients can be reversed by removing excess fat from liver and pancreas by means of calorie restriction [10,11].

Although various natural product-derived compounds demonstrate potent anti-diabetic activity, however to date, only a limited number of clinically-approved drugs such as metformin are used to treat T2DM [12]. Studies on the mechanism of action of these bioactive compounds demonstrated the important role of peroxisome proliferator activated receptor-gamma (PPARγ) in mediating their effects by increasing the sensitivity of peripheral tissues towards insulin and reducing blood glucose levels [12,13,14].

PPARγ, one of the three isoforms that belongs to a nuclear receptor PPAR family, plays a pivotal role in adipogenesis, lipid metabolisms and glucose homeostasis [15,16]. It acts as a transcription factor, which upon activation by its ligand, binds specifically to peroxisome proliferator responsive element (PPRE) present on the promoter of the target genes [17]. Thiazolidinediones (TZDs) which were clinically approved as drugs to treat patients with T2DM, have been identified as a full agonist of PPARγ [18] which upon activation regulates the expression of various genes responsible in reducing the insulin resistance in tissues—such as glucose transporter type 4 (GLUT4) and adiponectin [19]. Three synthetic TZDs have been FDA-approved for the treatment of T2DM, namely troglitazone, rosiglitazone and pioglitazone. However, diverse side effects elicited by these drugs such as cardiac problems, liver toxicity, fluid retention, edema, as well as bladder cancer in some patients, have led to the withdrawal of troglitazone from market in 2000 and use with caution for the other two TZDs [20,21]. Due to the growing concerns for diabetic patients that rely on TZDs, alternative drugs without major side effects sourced from natural products are highly anticipated.

The natural products have been used traditionally for thousands of years in the treatment of T2DM and plants are one of the most important sources of antidiabetic compounds. A review paper reported that 656 species from 437 genera, representing 111 plant families, with antidiabetic properties have been identified [22]. *Galega officinalis L.* (Fabaceae) was the first medicinal plant reported to have an antidiabetic effect and aguanidine derivative compound known as galegine, was isolated, which is responsible for lowering of blood glucose [23,24]. *M. charantia* or bitter gourd, belongs to the family Cucurbitaceae and originates in tropical Asia. It is characterised by warty-skinned fruit and widely cultivated in tropical and subtropical regions [25]. Parts of this plant have various medical properties such as fruit that are rich in vitamin C and phenolic compounds which have been shown to have anti-oxidant activities, while the leaf is used traditionally to treat stomach pain, anaemia, malaria, coughs and fever [26]. More importantly, studies have reported that the plant contained bioactive compounds with antidiabetic activities in in vitro and in vivo models, however, their role in activating PPARγ to induce insulin sensitisation in target tissues remains unexplored [27,28].

Therefore, in this study, THCB, a major cucurbitane triterpenoid compound isolated from *M. charantia* [29] was used to determine its effects in increasing the uptake of glucose in muscle cells by activating PPARγ as its ligand as well as inducing the expression and translocation of Glut4 to the cell membrane. The aim of these experiments are to determine the molecular mechanism behind the hypoglycaemic effect of this plant extract which is responsible for its anti-diabetic activity. Since *M. charantia* has a wide distribution of medicinal uses, it is imperative to postulate that it may act on the metabolic pathway such as those impacting on PPARγ molecule, at least in part. Hence, we chose to conduct further investigations in regard to PPARγ activation leading to hypoglycaemic-related effects.

## 2. Results

### 2.1. Momordica charantia Methanol Extract and THCB Induced PPRE Transcriptional Activity and May Function as PPARγ Ligand

Cell-based screening platform was used in this study to determine the natural products with potential activity as ligands for PPARγ. Various optimisation conditions were used such as the source of PPRE, transfection conditions and the presence or absence of expression plasmids of PPARγ2 and RXRα. PPRE originated from Acyl-Coenzyme A Oxidase (ACOX) gene, cells that were transfected in T75 flasks and subsequently transferred to 96-well plate for treatment together in the presence of expression plasmids of PPARγ2 and RXRα produced the most optimised and sensitive high throughput based screening platform (unpublished data).

**Figure 1:**
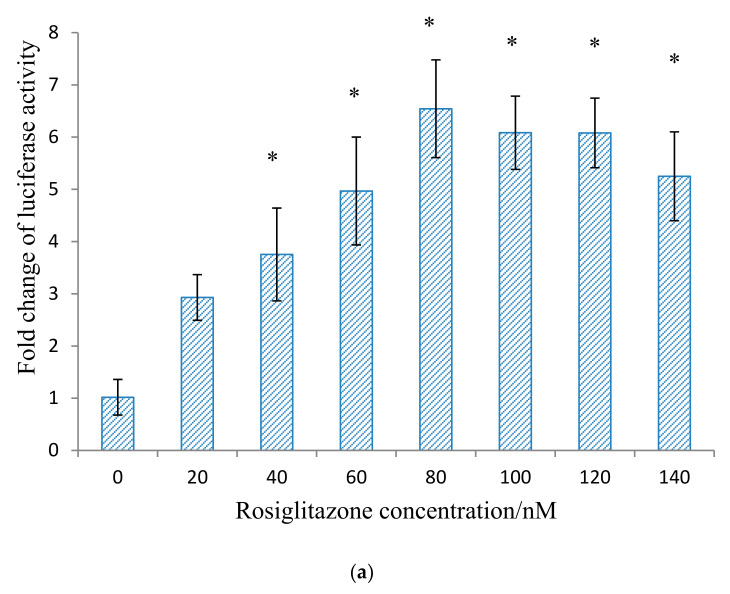

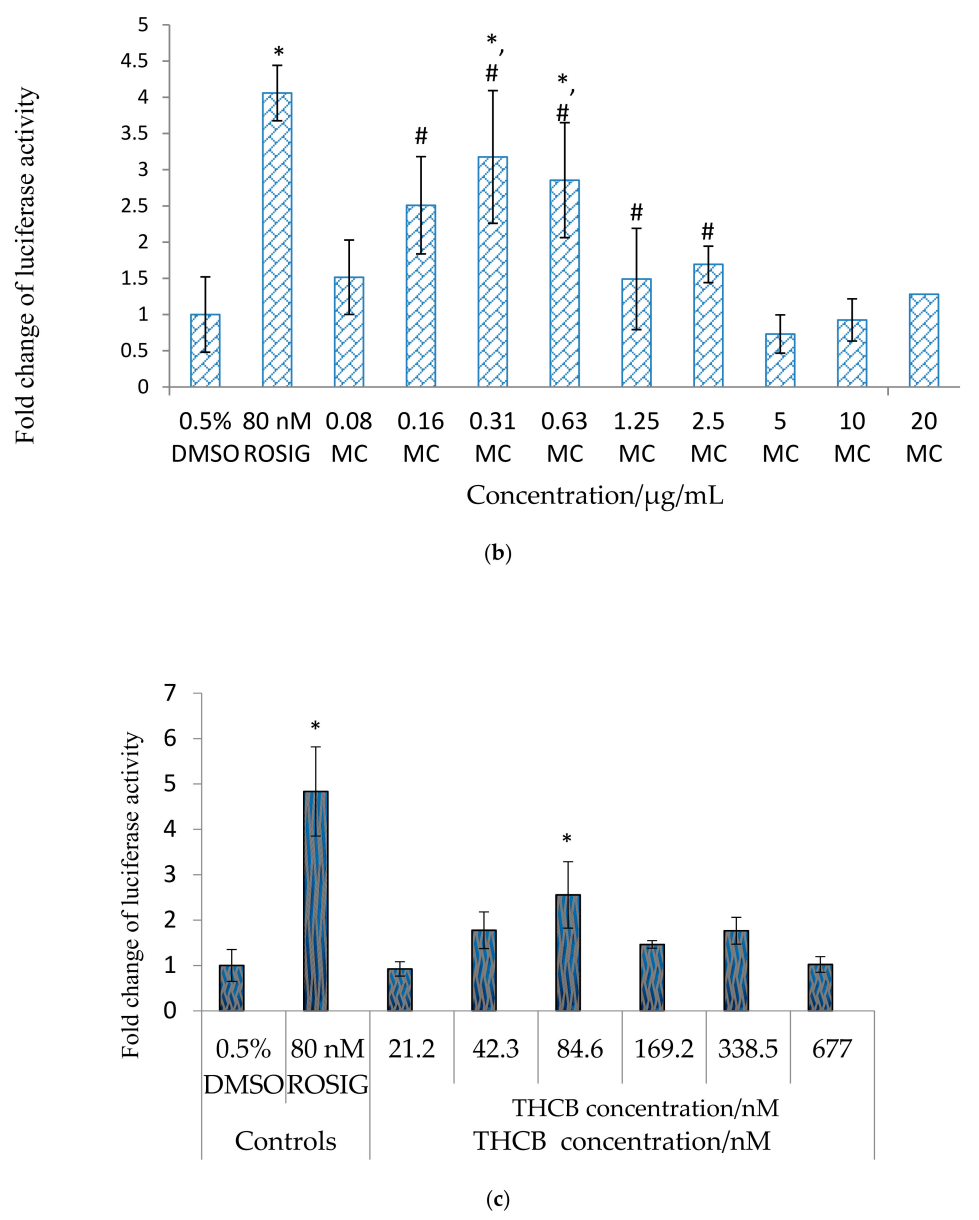
PPARγ ligand screening of (**a**) Rosiglitazone at concentrations ranging from 0–140 nM, (**b**) *Momordica charantia* (MC) fruit crude methanol extract from 0.08–20 μg/mL, (**c**) 3β,7β,25-trihydroxycucurbita-5,23(E)-dien-19-al (THCB) at concentration from 21.2–677 nM. HepG2 cells were transfected with PPRE-Tk-Luc, pSV-sport PPARγ, pSV-Sport RXRα, and pRL-CMV for overnight and treated as mentioned in the graph respectively. 80 nM rosiglitazone (Rosig) was used as the positive control, while 0.5% DMSO (which was used as carrier to dissolve the compound) was used as the negative control. After 23 h, luciferase activity was determined. Luciferase ratio of the cells treated with 0.5% DMSO was assigned to the value 1, and the results were presented as fold luciferase ratio relative to this value. Statistical analysis was conducted using one-way ANOVA (*p* < 0.05) where ‘*’ denotes statistically significant difference as compared to untreated control, while ‘#’ denotes not statistically different when compared to positive control rosiglitazone where applicable.

As shown in Figure 1a, when the developed cell-based screening platform utilising the cells transfected with conditions described in methodology, and were treated with various concentrations of rosiglitazone, there was a significant dose-dependent increase of transcriptional activity of PPRE until it reached its highest level at 80 nM where the activity was increased by 7-fold as compared to untreated control. However, there was no significant change in transcriptional activity when the cells were treated from 80 to 140 nM rosiglitazone indicating that a saturation level was reached at 80 nM.

Subsequently, methanol extract from *M. charantia* fruit was subjected to cell-based assay to determine the presence of the potent PPARγ ligands. It was found that the transcriptional activity of PPRE significantly increased when the transfected cells were treated with 0.16 μg/mL extract, reached its peak at 0.31 μg/mL (increased by 3-fold) and declined at 0.63–2.50 μg/mL but remained significantly higher as compared to that of untreated control, as shown in Figure 1b. The luciferase activity was further decreased and not significantly different as compared to untreated control when the cells were incubated with the extract at 5 μg/mL and higher.

Based on literature search, 228 different medicinal compounds have been identified from *M. charantia* [30] and a compound known as 3β,7β,25-trihydroxycucurbita-5,23(E)-dien-19-al (THCB) which is a cucurbitane triterpenoid was demonstrated to produce hypoglycaemic effect in mice [29]. However, the target of this compound in exerting its antidiabetic effect in mice was unknown. Therefore, it was important to determine on the role of THCB as the candidate for PPARγ ligand. As demonstrated in Figure 1c, transfected cells treated with THCB at concentrations 21.2–677 nM showed significantly higher transcriptional activity than to that of untreated control cells at 84.6 nM but not at other concentrations used.

### 2.2. THCB Bound within the Ligand Binding Domain of PPARγ Mostly via Non-Polar Interactions

**Figure 2:**
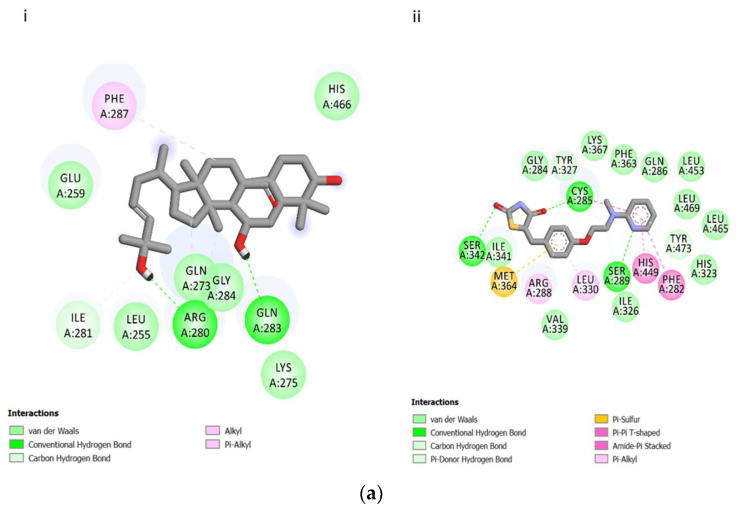

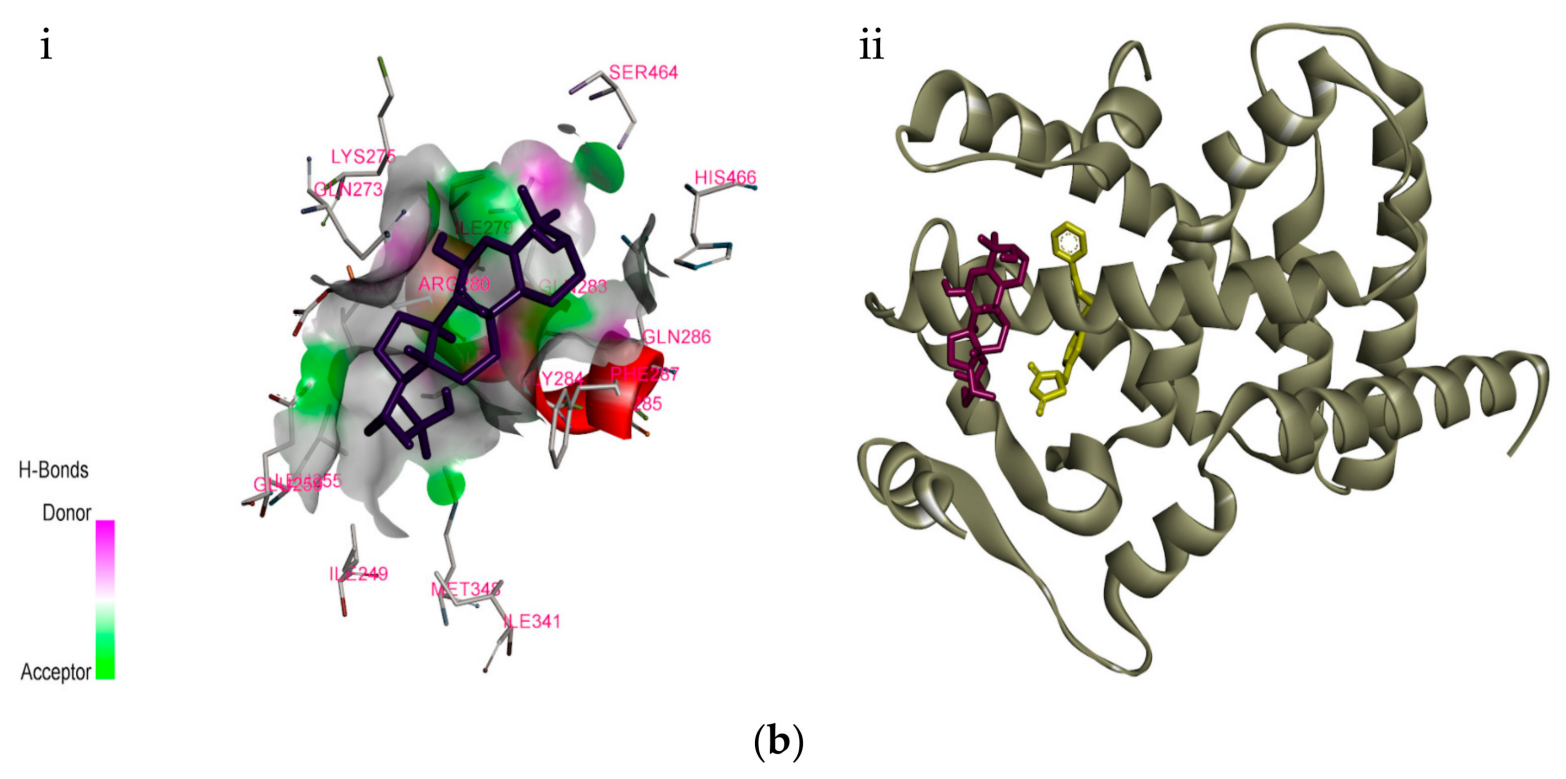
(**a**) 2-Dimensional binding interaction of THCB (i) and rosiglitazone (ii) with PPARγ nuclear receptor. Diagram was generated from Discovery Studio 4.1. Green residues represent non-polar contact while purple residues represent polar contact. The types of interactions are specified under each figure. (**b**) Interaction diagrams generated from Discovery Studio 4.1 represent the binding mode of THCB in the ligand binding domain of PPARγ (i), and the molecular view both rosiglitazone (yellow) and THCB (purple) in the active site of PPARγ (ii).

Molecular docking was conducted to elucidate the mode of binding of THCB within PPARγ as well as the type of interactions involved. The 2D structures as well as binding interactions of rosiglitazone and THCB with PPARγ are shown in Figure 2a. The binding mode of THCB in the ligand binding domain of PPARγ was examined in depth using molecular docking analysis. The analysis of docking indicated that THCB has a lower binding affinity (with binding energy of −7.7 kcal/mol) in the ligand binding domain of PPARγ compared to rosiglitazone (with binding energy −8.0 kcal/mol). The binding interactions of THCB was analysed and compared with binding interactions of the control rosiglitazone. With reference to Table 1, as compared to rosiglitazone, THCB results in lower binding site interactions with the amino acids of PPARγ. The number of hydrogen and van der waals bonding between THCB with amino acids that contributed to the binding with PPARγ (Figure 2(ai)) were also lesser compared to those resulted with rosiglitazone as demonstrated in Figure 2(aii). Most of the interactions occurred with THCB binding are of non-polar nature, whereas those of rosiglitazone are comprised of both polar and non-polar. All of these factors contribute to the lesser binding affinity of THCB as compared to rosiglitazone towards PPARγ.

Figure 2b(i) depicts the interaction diagram which is representing the binding mode of THCB within the PPARγ, while Figure 2b(ii) portrays both THCB and rosiglitazone within the ligand binding domain of PPARγ, showing clearly that the position of the respective compounds are not overlapping. Hence, these results suggest that THCB does not exhibit similar binding modes with the potent PPARγ agonist rosiglitazone.

### 2.3. Insulin, Rosiglitazone and THCB Induced 3T3-L1 Pre-Adipocytes Differentiation to Mature Adipocytes

**Figure 3:**
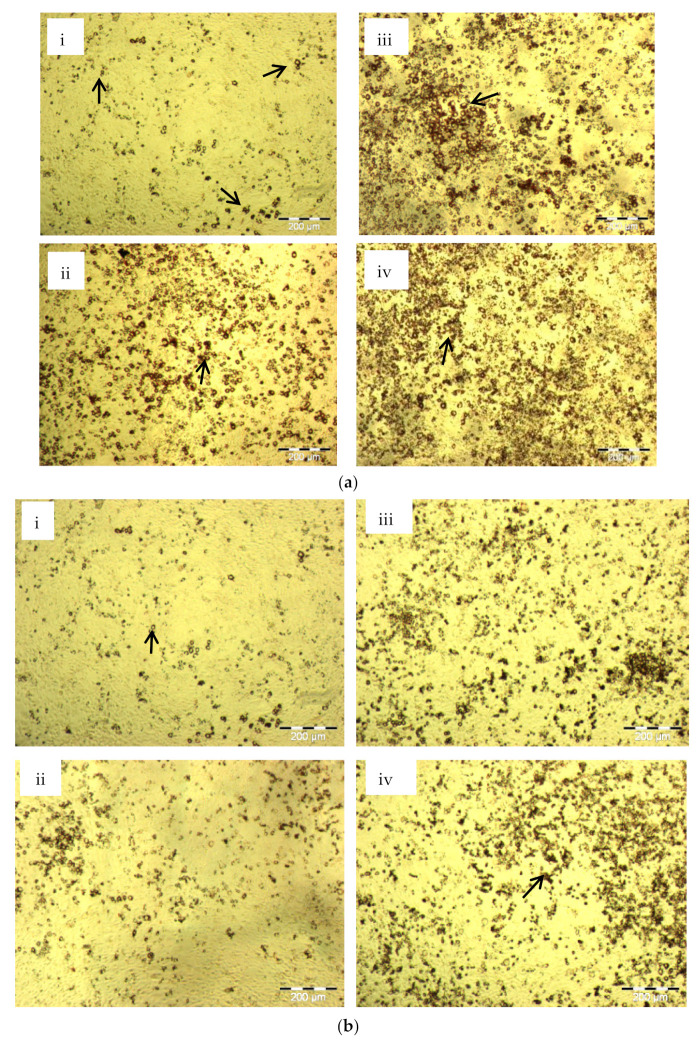

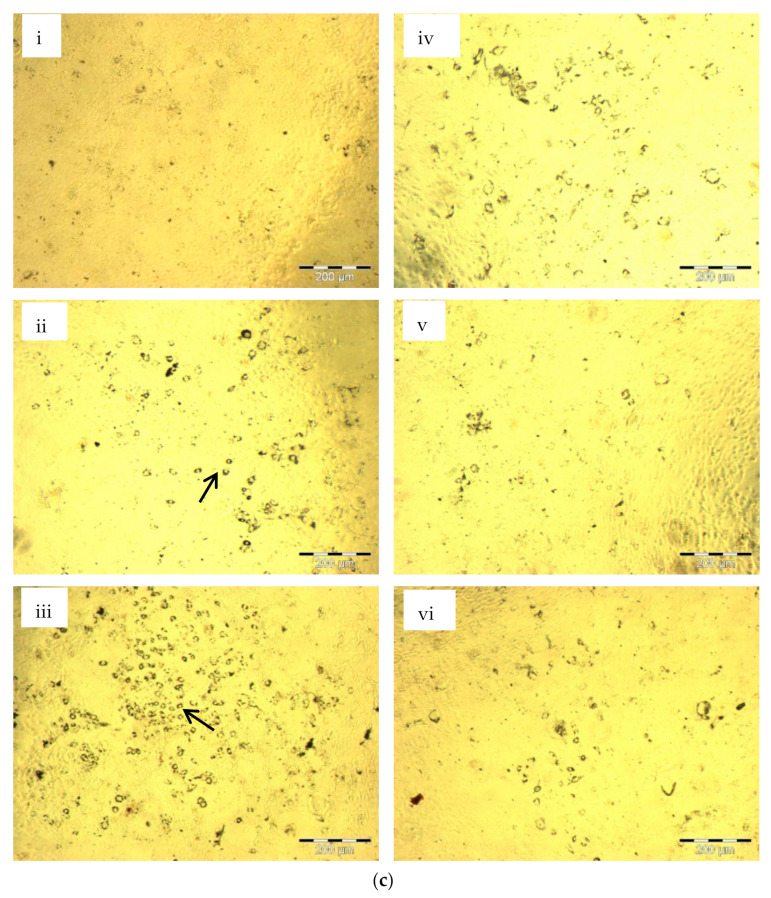
(**a**) Insulin induced 3T3-L1 pre-adipocytes differentiation to mature adipocytes. 3T3-L1 pre-adipocytes were induced to differentiate in complete medium containing 10%FBS supplemented with IBMX-Dexamethasone (Dex) during day 2 post-confluence, without insulin (i), or using 0.17 µM insulin (ii), 1.74 µM insulin (iii) or 17.4 µM insulin (iv). Oil droplets were stained in dark red using oil red-O staining, illustrated by small arrows in the figures. A dose-dependent increase in oil droplets formation was observed when there was an increase in insulin concentrations. (**b**) Rosiglitazone induced 3T3-L1 pre-adipocytes differentiation to adipocytes.3T3-L1 pre-adipocytes were induced to differentiate during day 2 post-confluence up to day 8, using 0 µM (i), 0.01 µM (ii), 0.1 µM (iii), 1 µM (iv), rosiglitazone in complete medium containing 10% FBS, supplemented with IBMX-Dex. A dose-dependent increment in oil droplets formation was evident. Oil droplets were stained in dark red using oil red-O staining and indicated by small arrows in the figure. (**c**) THCB supported 3T3-L1 pre-adipocytes differentiation to mature adipocytes.3T3-L1 pre-adipocytes were induced to differentiate during day 2 post-confluence up to day 8, using 0 nM (i), 106 nM (ii), 212 nM (iii), 423 nM (iv), 846nM (v) and 1690 nM (vi) THCB in IBMX-Dex medium. Oil droplets were stained in dark red using oil red-O staining and indicated by small arrows in the figure. Oil droplets were evident by THCB treatment at 106–423 nM. All figures are at 40× magnification.

As shown in Figure 3a, there was an increase in the number of 3T3-L1 pre-adipocytes differentiated into mature adipocytes when the cells were incubated with increased concentrations of insulin. In contrast, most of the cells were not differentiated into mature adipocytes in the absence of insulin. Similarly, rosiglitazone at concentrations ranging from 0.001 to 10 μM, also induced fat cell differentiation in dose-dependent manner (Figure 3b).

Based on cell-based screening assay, THCB was identified as a potent PPARγ ligand. Therefore, in order to determine the function of THCB in mimicking the ability of rosiglitazone in inducing the differentiation of 3T3-L1 preadipocytes to mature adipocytes, the cells were treated with IBMX-Dex in the presence of various concentrations of THCB. There was a dose-dependent increase in the formation of lipid droplets indicating preadipocytes were differentiated into mature adipocytes when the cells were treated with 106 nM, 212 nM and 423 nM (Figure 3c(ii–iv)). The number of cells differentiated was smaller in THCB-treated cells than compared to that of insulin and rosiglitazone-treated cells. Interestingly, when treated at higher concentrations than 212 nM, the number of mature adipocytes gradually decreased. The results strongly suggested that THCB was not a potent inducer of adipocytes differentiation and only supported adipocytes differentiation at lower concentrations (106 nM up to 423 nM).

### 2.4. THCB Regulated PPAR Gamma Target Genes in Differentiated 3T3-L1 Cells

In order to further characterise THCB as a potent PPARγ ligand, the effects of the compound on the expression levels of PPARγ target genes were determined in differentiated 3T3-L1 cells. The selected PPARγ target genes were lipoprotein lipase (LPL) which is involved in lipid metabolism [31] and adipocyte fatty acid binding protein (AP2) which is involved in uptake and metabolism of fatty acids as well as the modulation of specific enzymes of lipid metabolism, cell growth and differentiation [32], adiponectin which is closely associated with insulin sensitivity [33], and, fatty acid translocase (CD36), a type of membrane fatty acid handling proteins [34].These genes were also used by Jeong et al. [35] to study the induction of PPARγ target genes by its ligands in 3T3-L1 adipocytes. In the gene expression experiment, THCB was used at 212 nM because this compound has shown the highest adipocyte differentiation activity at this concentration, as demonstrated in Figure 3c.

**Figure 4:**
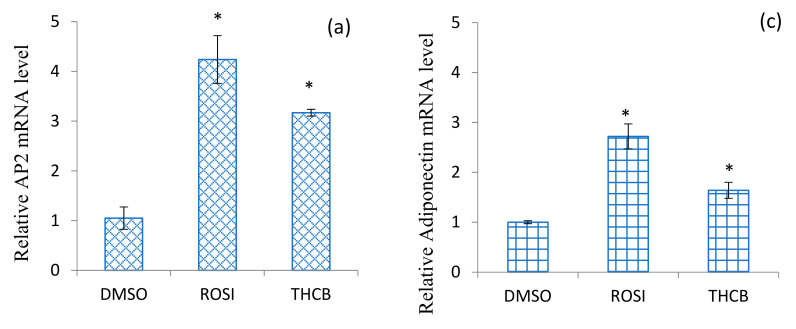

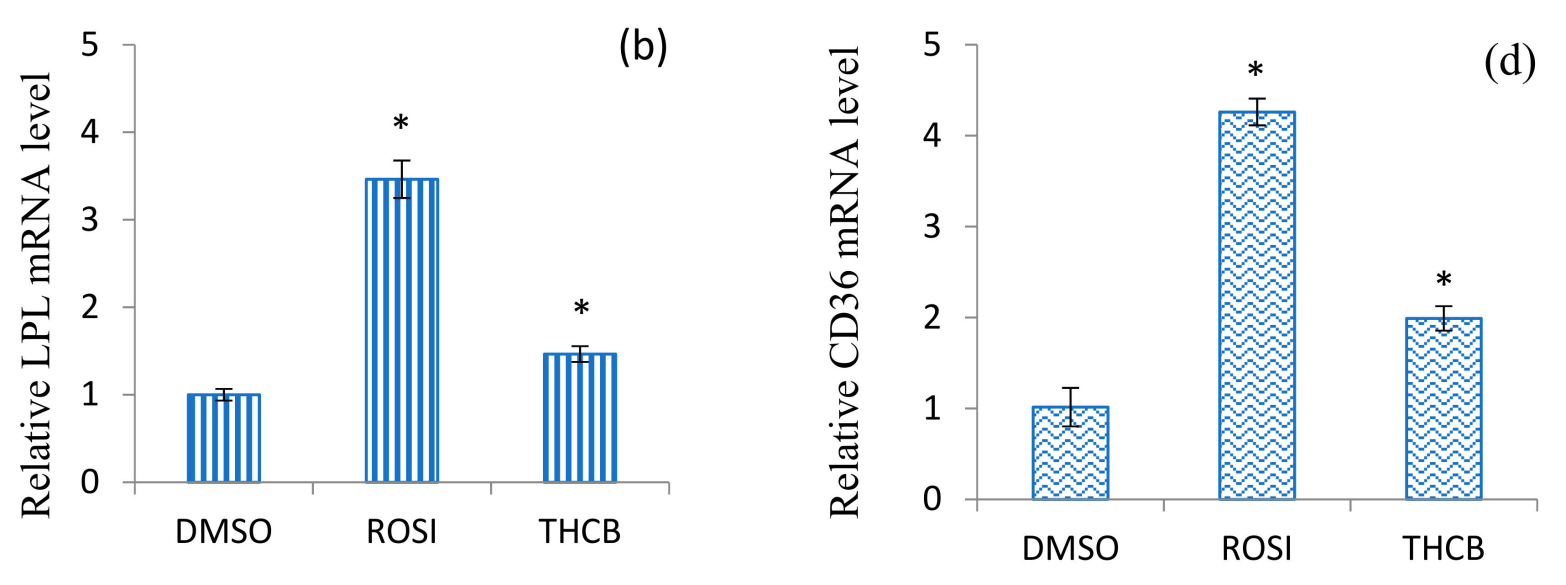
Effect of THCB compound on the expression levels of PPARγ-target genes in 3T3-L1 adipocytes. 3T3-L1 adipocytes were incubated for 24 h with either 0.5% DMSO (DMSO), 100 nM rosiglitazone (ROSI) or 212 nM THCB. The mRNA expression levels were normalised to β-Actin mRNA for each sample. Each bar represents mean ± SEM of experiments conducted in triplicate. * indicates *p* < 0.05 than the negative control when evaluated using paired t-test. PPARγ-target genes tested were (**a**) AP2, (**b**) LPL, (**c**) Adiponectin, (**d**) CD36.

Figure 4 demonstrates that when 3T3-L1 adipocytes were treated for 24 h with 212 nM THCB, generally there was a significant increase in the expression levels of AP2, adiponectin, LPL and CD36 genes. THCB increased the levels of AP2 mRNA expression by 3.2 ± 0.07 fold, adiponectin by 1.64 ± 0.16 fold, LPL by 1.5 ± 0.09 and CD36 by 2.0 ± 0.14 fold. Similarly, treatment with potent PPARγ ligand rosiglitazone also produced a significant increase in those four genes with expression levels of AP2, adiponectin, LPL and CD36 were upregulated by 4.2 ± 0.5, 2.7 ± 0.3, 3.5 ± 0.2 and 4.3 ± 0.2 folds, respectively. The results clearly indicate that THCB exhibited similar pattern of upregulation of AP2, adiponectin, LPL and CD36 mRNA levels as rosiglitazone, which in turn, suggest the potent role of the compound as PPARγ ligand.

### 2.5. THCB Induced Glucose Uptake in C2C12 Myotubes

**Figure 5:**
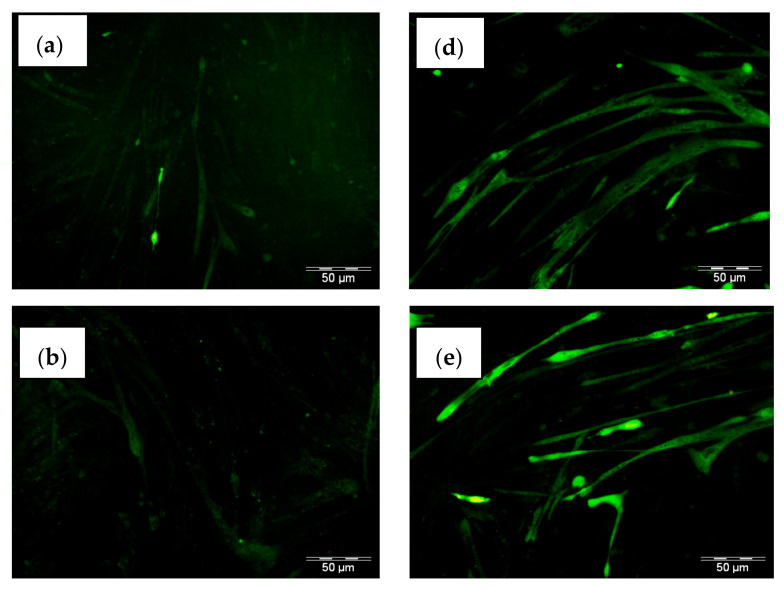

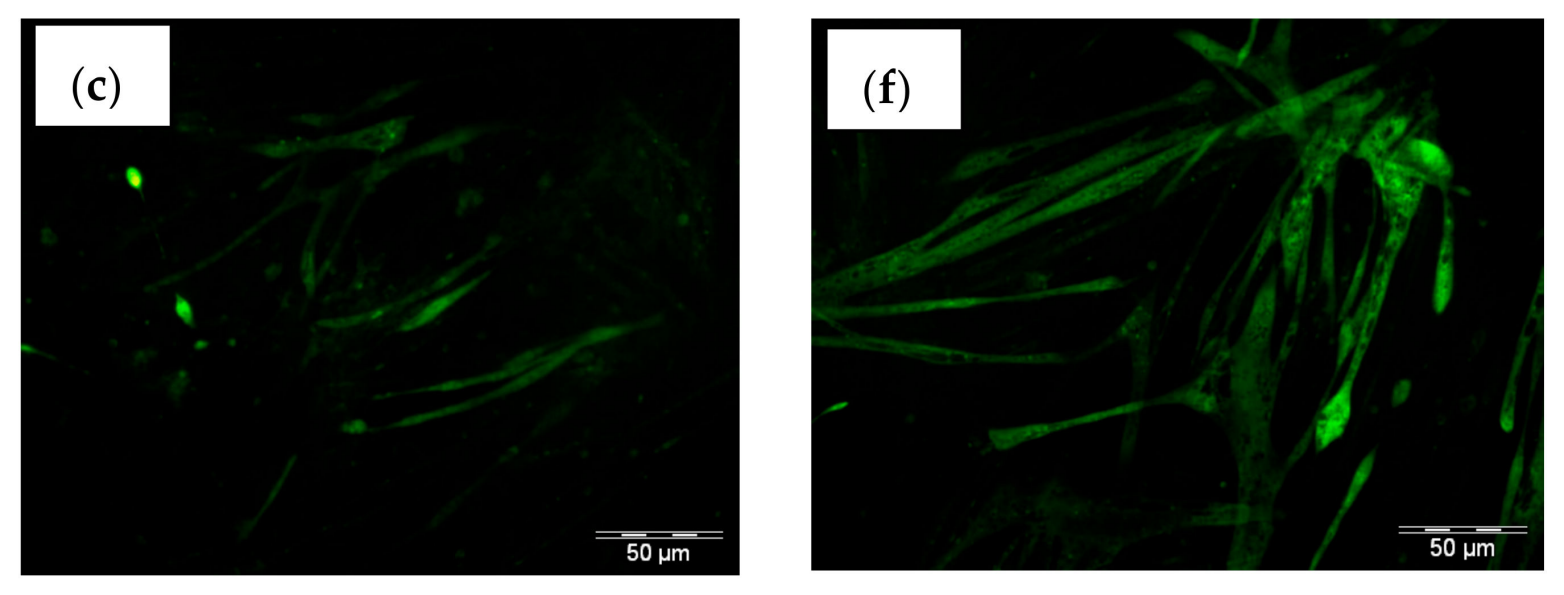
The effect of THCB on glucose uptake in muscle cells. C2C12 myotubes were treated for 24 h with (**a**) 1% DMSO as negative control, (**b**) 1.7 µM THCB, (**c**) 3.3 µM THCB, (**d**) 6.6 µM THCB, (**e**) 13.2 µM THCB, and 2 h treatment for 17.4 µM Insulin (**f**) as the positive control, before subjecting to 2 NBDG. Figures are 100× magnifications, each represent one of three independent experiments with similar results.

In order to determine the effects of THCB as the natural PPARγ ligand in inducing glucose uptake in muscle cells, C2C12 myotubes was selected as the model system. As shown in Figure 5, it was demonstrated that the treatment of C2C12 myotubes with insulin produced an increase in glucose uptake as compared to control. Interestingly, there was a dose-dependent increase in glucose uptake by C2C12 myotubes when the cells were treated in increasing concentrations of THCB starting from 1.7 µM up to 13 µM, indicating that THCB may induce glucose uptake via the activation of PPARγ by acting as its ligand. In this experiment, THCB concentration was extended to higher concentration levels as compared to other experiments to observe the effect of glucose uptake in C2C12 myotubes at higher concentration range.

### 2.6. THCB Increased the Translocation of GLUT4 Protein in C2C12 Myotubes

**Figure 6:**
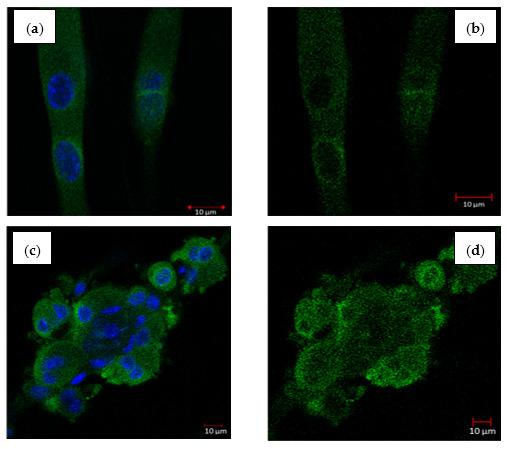

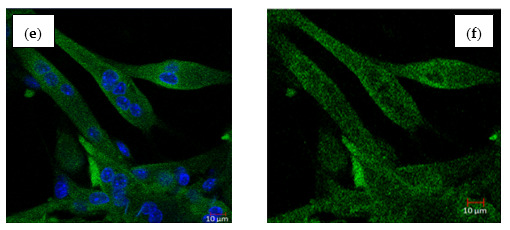
The effect of THCB on Glut 4 translocation in muscle cells. C2C12 myotubes were treated for 24 h with (**a**) 0.5% DMSO as negative control, nuclei were labelled with DAPI, (**b**) 0.5% DMSO as negative control, nucleus not captured (**c**) 1 µM THCB, nuclei were labelled with DAPI, (**d**) 1 µM THCB, nucleus not captured (**e**) 2 µM THCB, nuclei were labelled with DAPI, (**f**) 2 µM THCB, nucleus not captured. The fluorescent images were captured using 63× oil immersion objective of LSM710 laser scanning microscope (Carl Zeiss, Oberkochen, Germany). Figures represent one of three independent experiments with similar results.

PPARγ was shown to induce glucose uptake in muscle cells and adipocyte via GLUT4 receptors present on the cell surface [36,37]. Rosiglitazone which is a potent PPARγ agonist has been demonstrated to induce the activity of PPARγ and GLUT 4 protein level in the muscles of diabetic-induced obese rats by streptozotocin [38]. α-Mangostin, a compound from *G. malaccensis* stimulates glucose uptake via increased in Glut 4 and leptin protein expression [39]. Bone/body morphogenetic proteins (BMP) are growth factors involved in inducing bone and cartilage development, and, BMP2 and BMP6 act on mature adipocytes as insulin-sensitizers. As revealed by whole transcriptome analysis, BMP signalling leads to transcriptional upregulation of several genes involved in lipid metabolism, including PPARγ and GLUT4 [40]. Figure 6 shows the effects of THCB in regulating the translocation of GLUT4 to the cell membrane in C2C12 myotubes. It was found that 1 µM and 2 µM THCB induced the translocation of GLUT4 protein from an area surrounding nuclei and cytoplasm to the cell membrane.

## 3. Discussion

Diabetes mellitus is the number ninth cause of death in the world and T2DM contributed to 90% of the total cases of this metabolic disease [41]. One of the hallmarks of T2DM is insulin resistance and currently TZD is one of the first options of oral drug being recommended to manage T2DM patients, besides metformin and sulfonylureas [42]. It was widely reported that TZDs function by increasing the insulin sensitivity and glucose uptake in liver, adipocytes and muscle cells [42,43,44]. More importantly, TZDs were demonstrated to serve as the ligands for PPARγ, a transcription factor that is responsible in regulating the transcriptional regulation of genes involved in glucose and lipid homeostasis [45].

Glut4 is the main receptor present on the surface of adipocytes and skeletal muscles, which upon insulin induction, increases the uptake of glucose from the blood into these cells. Various binding sites for transcription factors were identified in rat and human promoter region of Glut4 that mediate the action of insulin [46]. Interestingly, PPRE, a binding site of PPARγ, was present in Glut4 promoter and treatment of adipocytes with rosiglitazone induced the transcriptional activity of GLUT4 promoter via detaching PPARγ from the gene promoter, resulting in increased Glut 4 expression and more importantly enhanced insulin sensitivity [36]. Other than augmenting Glut 4 expression in differentiating adipocytes, rosiglitazone also improves Glut 4 translocation both in differentiating as well as fully differentiated adipocytes [47].

*M. charantia* fruit is a popular vegetable which is widely known to have various health beneficial effects especially in the treatment of T2DM [48,49]. However, to date, there is still a lack of systematic study investigating the mechanisms of action of the compounds isolated from the plant in inducing glucose uptake in skeletal muscles. Therefore, in this study, the action THCB, a compound known to be present in *M. charantia*, as potential ligand of PPARγ in inducing Glut4 translocation to the cell membrane as well as increasing glucose uptake was elucidated.

Based on cell-based screening assay utilising PPRE-linked reporter system, the methanol extract contained compounds may serve as the potential ligand for PPARγ (Figure 1b). Other studies also reported that extract preparations from hexane, ethyl acetate, water of *M. charantia* induced PPARγ activity [50,51]. In addition, n-butanol layer of methanol extract (extract P) and n-butanol layer of methanol extract without n-hexane or ethyl acetate soluble compounds (extract G) reduced the blood glucose levels in insulin-resistance rats via the activation of PPARγ which clearly indicates the presence of compounds representing its ligands [52].

From the molecular docking analysis, THCB has lower binding interactions, hence lower binding affinity towards PPARγ as compared to rosiglitazone. This lower binding affinity of THCB within the ligand binding domain of PPARγ observed from our molecular docking analysis can be associated with the lower effects of THCB on PPRE activation, target gene expression as well as adipocyte differentiation as compared to those induced by rosiglitazone. Moreover, we have demonstrated that the mode of binding is not overlapping between THCB and rosiglitazone within PPARγ ligand binding domain, which explains the difference in potency.

Although there are many studies carried out on the determination of the anti-diabetic effects of extracts prepared from *M. charantia*, research on isolated compounds is more limited. To date, only a few compounds isolated from the plant were shown to possess activity as PPARγ ligands, such as 3β,7β-dihydroxy-25-methoxycucurbita-5,23-diene-19-al (DMC) and a cucurbitane-type triterpene glycoside known as compound 13 [53,54]. In our study, THCB, a compound previously isolated from *M. charantia* was found to activate the PPRE transcriptional activity suggesting its potential role as PPARγ ligand. It was reported that THCB has similar structure as compound 13 [54] and induced hypoglycaemia in mice [29] which further supported the ability of the compound to bind and activate the transcription factor.

Different ligands have their own affinities for PPARγ, for example TZDs have affinity towards PPARγ in the range 40–700 nmol/L [55]. Other ligands such as linoleic acid, 15-d-PGJ2, indomethacin and palmitic acid have binding affinities in the range of 1.3–4.9, 11.6–15.1, 38–42, and 156 μM towards PPARγ ligand binding domain respectively [56]. In addition, TZDs were reported to have PPARγ independent actions at very elevated concentrations, including activation of other nuclear hormone receptors [57]. These facts could be associated with the decrease in transcriptional activity of PPRE during treatment with rosiglitazone and THCB after certain concentrations, as observed in our experiments.

Between the methanolic extract of *M. charantia* and its compound THCB, the former induced higher magnitude of PPRE transcriptional activity in our experiment. This could be owing to the fact that in the extract form, other than THCB, there are many other compounds present in *M. charantia* which have been demonstrated to have in vivo hypoglycaemic activity in diabetic mice including another cucurbitane triterpenoid with the name 5β,19-epoxy-3β,25-dihydroxycucurbita-6,23(E)-diene [29], charantin, polypeptide-p and vicine [58]. Ironically, sitosterol and stigmastadienol glycosides which are two aglycones of charantin, did not produce hypoglycaemic activity when tested in vivo separately [58]. Hence, based on our results which are also supported by these findings, we postulate that there is synergistic activity of the phytochemicals present in the *M. charantia* extract in driving PPRE transcriptional activity.

Upon binding by its ligands, PPARγ regulates the expression of key genes that are responsible in modulating adipocyte differentiation as well as lipid and glucose homeostasis [59,60,61]. Fatty acid binding protein (AP2) is a marker commonly used to mark differentiated adipocytes and expressed in the stromal vascular fraction (or progenitor cells) of white and brown adipose tissues [62]. Similarly, overexpression of adiponectin in 3T3-L1preadipocyte enhanced the cells proliferation, accelerated adipocyte differentiation as well as induced lipid accumulation and more importantly increased glucose uptake via Glut4 [63]. Fatty acid translocase, CD36, a membrane glycoprotein found on adipocytes [64], is responsible in lipid utilisation, adipose energy storage, fat absorption and long-chain fatty acids transportation across the plasma membrane into cells [65]. Lipoprotein lipase, the other key enzyme involved in lipid metabolism, plays an important role in regulating lipid content by hydrolysing triacylglycerol component of chylomicron and VLDL into free fatty acids [66,67]. It was widely demonstrated that the expression levels of these genes in matured adipocytes are correlated with increase in PPARγ expression [68,69]. It is consistent with the results obtained from our study where THCB increased the expression of all these four genes (Figure 4) albeit at the levels lower than rosiglitazone, which may support that THCB is playing a role as PPARγ agonist.

This is further supported with the ability of THCB to induce adipocyte differentiation although the number of pre-adipocytes differentiated into matured adipocytes was lower than to that induced by rosiglitazone (Figure 3b,c). GW-0072 and indomethacin which are known PPARγ partial agonists also elicited a lower increase in mRNA levels of genes involved in lipid metabolism and differentiation as compared to that induced by pioglitazone [70]. In addition, lower intracellular lipid accumulation was observed when both pre-adipocytes and mesenchymal stem cells were differentiated to adipocytes using these partial agonists as compared to thiazolidinediones [70,71].

It was widely demonstrated that PPARγ activation regulates the expression of various genes which subsequently induces glucose uptake in adipocytes and muscle cells [72]. Previous studies showed various extracts and compounds of *M. charantia* produced an increase in glucose uptake in both adipocytes and muscle cells via the activation of PPARγ which clearly indicates the presence of agonists in the plant [50,51,52,73]. This is in agreement with results obtained in this study where the glucose uptake was increased in dose-dependent manner when the cells were treated with various concentrations of THCB. One of the mode of action of PPARγ in increasing insulin sensitivity is via increasing the expression of adiponectin [74]. In addition, insulin sensitivity was reported to be linked to adiponectin sensitivity index, which is derived from adiponectin secretion from adipocytes due to treatment with PPARγ agonist such as TZD [75]. Moreover, Glut4 presence on the cell surface was shown to play an important role in transporting and taking up circulating glucose into the cells and tissues [76]. THCB was demonstrated to increase the expression of adiponectin in matured adipocytes which strongly suggests that THCB may act via this mode of action. Interestingly, treatment of C2C12 muscle cells with THCB also increased the translocation of Glut4 to the cell membrane indicating that THCB as PPARγ ligand activated the receptor which in turn increased the presence of Glut4 on the cell surface to induce the uptake of glucose. PPARγ synthetic and natural ligands were shown to induce glucose uptake by increasing the expression and translocation of Glut4 to the plasma membrane of both adipocytes and muscle cells [28,77]. Although, *M. charantia* exerted its effects in inducing the glucose uptake via both insulin-dependent (IRS-PI3K) [78] and insulin-independent (AMPK pathways) [79], it is tempting to speculate that THCB induced the glucose uptake by increasing the number of Glut4 on cell surface via the activation of PPARγ and insulin sensitivity. In addition, since THCB did not produce higher adipogenic activity which induced lower number of adipocytes differentiation, the compound may offer less side effects in reducing the risk of obesity as compared to TZDs [80].

## 4. Materials and Methods

### 4.1. Materials

HepG2 hepatocytes, 3T3-L1 fibroblasts and C2C12 myoblasts were purchased from American Type Culture Collection (ATCC, Rockville, MD, USA). 3β,7β,25-trihydroxycucurbita-5,23(E)-dien-19-al (THCB) was isolated from *Momordica charantia* by Wuxi Apptec, Shanghai, China. Gibco high glucose Dulbecco’s modified eagles medium (DMEM), Gibco Opti-MEM reduced serum medium (RSM), Gibco Minimum essential media (MEM), fetal bovine serum (FBS), bovine serum (BS), horse serum (HS), Lipofectin Transfection Reagent and penicillin and streptomycin were from Life Technologies (Grand Island, NY, USA). 0.075% sodium bicarbonate and sodium pyruvate were from Sigma Aldrich. 2-(N-(7-Nitrobenz-2-oxa-1,3-diazol-4-yl)Amino)-2-Deoxyglucose(2-NBDG) were from Molecular Probes (Grand Island, NY, USA). PPREx3-TK-Luc (Addgene plasmid # 1015), pSV-sport PPARγ2 (Addgene plasmid # 8862) and pSV-sport RXRα (Addgene plasmid # 8882) plasmids were provided by Bruce Spiegelman, and pRL-CMV was purchased from Promega Corporation (Madison, WI, USA).

### 4.2. Extraction of Momordica charantia

A total of 2 kg of fresh *M. charantia* fruits was purchased from a local market in Penang, Malaysia. Sample of the plant part was deposited and identification was carried out by a botanist in Universiti Sains Malaysia (USM) Herbarium with voucher specimen number 11522. The fruits were sliced into small pieces and dried in drying oven at 50 °C for 3–5 days. Following that, the dried plants were ground in a grinder, soaked with methanol for 2 h and filtered through a filter paper (Whatman International Ltd., Maidstone, Kent, England). Methanol extract was then dried in a rotary evaporator and dissolved in DMSO at 2 mg/mL.

### 4.3. Mammalian Cell-Based PPARγ Ligand Screening Assay

Complete MEM was prepared by supplementing MEM with 2 mM L-glutamine medium with 1× MEM non-essential amino acids solution, 1 mM sodium pyruvate and 1× penicillin and streptomycin. HepG2 cells were seeded in T75 and cultured overnight in complete MEM + 10% FBS to achieve 80% confluency. The cells were then transiently transfected with a transfection cocktail containing four plasmids according to manufacturer’s protocols. Briefly, Lipofectin working solution was prepared by adding 22.5 µL Lipofectin into 727.5 µL RSM and incubated for 30 min in a sterile 1.5 mL eppendorf tube. In another 1.5 mL eppendorf tube, a plasmid cocktail was prepared by mixing 24 µg PPREx3-TK-Luc, 24 µg pSV-sport PPARγ2, 24 µg pSV-sport RXRα and 0.6 µg pRL-CMV, and RSM was added into a final volume of 750 µL. Then, the contents of both tubes were mixed and incubated for a further 10 min. Subsequently, 6 mL RSM was added into the mixture to form 7.5 mL transfection solution.

Culture medium in T75 flask was discarded and the transfection solution was overlaid on cells followed by overnight incubation. Subsequently, the cells were trypsinised and subcultured into two units of 96-well white clear bottom plates in 50 μL medium for each well. Rosiglitazone or *M. charantia* extract was then added at various concentrations in 50 μL volume and the plate was incubated for 24 h. Then, the luciferase activity was determined using Dual-Glo Luciferase Assay System (Promega Corporation, Madison, WI, USA), according to the manufacturer’s instructions.

### 4.4. Determination of the Mode of Binding and Interaction Types of THCB and PPARγ Using Molecular Docking

The Protein Data Bank (PDB) structure of Human PPARγ ligand binding domain complexed with rosiglitazone was obtained from Discovery Studio Client (Version 4.1 Dassault Systèmes BIOVIA, Marcel Dassault, Paris, France) software with PDB ID:5YCP [81]. This software was utilised for viewing and analysing the binding sites. Using PDB ID:5YCP, PPARγ protein structure was separated from its ligand complex, followed by performing docking experiment using AutoDock Vina 1.1.2 (La Jolla, CA, USA) [82] and Autodock Tools 1.5.6 (La Jolla, CA, USA) [83] protocols at the ligand binding domain of PPARγ. Control docking experiment was performed using rosiglitazone for validating the docking result. Following this, THCB was docked into the PPARγ protein structure to determine the point where the lowest free energy of binding occurs. The lowest free energy value indicates the most favourable binding towards PPARγ.

### 4.5. Adipocyte Differentiation

3T3-L1 preadipocytes were cultured in DMEM with L-glutamine (4 mM), and supplemented with 0.075% (*v*/*v*) sodium bicarbonate, 1 mM sodium pyruvate, 1× penicillin and streptomycin (complete medium) with 10% BSin 24-well plates. After 2 days post-confluence (designated as day 0 of differentiation), the cells were differentiated to adipocytes in the presence of 0.5 mM IBMX, 0.5 µM dexamethasone and 0.85 µM insulin in DMEM with 10% FBS (MDI medium). In order to see the effect of THCB in adipocyte differentiation, 0.85 µM insulin was replaced with THCB compound at various concentrations during treatment with MDI medium. After 2 days of incubation, the medium was changed to fresh complete medium with 10% FBS in addition to 0.85 µM insulin or THCB at various concentrations. At day 5, the medium was replaced with fresh complete DMEM with 10% FBS until day 8. Subsequently, the cells were rinsed with PBS and fixed with 4% paraformaldehyde for 30 min, washed with PBS and rinsed briefly with 60% isopropanol, followed by staining with Oil Red O in 60% isopropanol to stain for lipid droplets for 1 h. Cells were then washed with fresh 60% isopropanol followed by rinsing 3 times with PBS. This protocol was from Lillie and Ashburn [84] with some modifications. Then, the cells were viewed under phase contrast, inverted microscope and images were captured.

### 4.6. Gene Expression Analysis

Real-time PCR was conducted using iScript One-Step RT-PCR Kit (Hercules, CA, USA) with SYBR Green in BIO-RAD iQ5 iCycler. Reaction was prepared in a total of 30 µL reaction mix which composed of 15 µL 2× SYBR Green RT-PCR Reaction mix, 200 nmol of forward and reverse primers respectively, 100 ng total RNA, 0.5 µL iScript Reverse Transcriptase for One-Step RT-PCR and ddH_2_O was added to a final volume of 30 μL. The following programme was used to amplify the genes: 10 min at 50 °C and 5 min at 95 °C followed by 30 cycles of 95 °C for 10 s and 54 °C for 30 s. Finally, the reaction was completed by the melt curve analysis which consisted of incubation for 1 min at 95 °C, 1 min at 55 °C, and 10 s at 55 °C for 80 cycles, with an increase of 0.5 °C after each cycle.

### 4.7. Myotubes Differentiation

C2C12 myoblasts were maintained in DMEM supplemented with 10% FBS. For myotubes differentiation, myoblasts were cultured in 24-well plate until they reach confluency and the medium was replaced with DMEM supplemented with 2% HS for 6–8 days. The medium was refreshed every 2 days during differentiation period.

### 4.8. Glucose Uptake Assay

C2C12 myotubes were treated with THCB or insulin for 24 h and 2 h, respectively. After the treatments, C2C12 myotubes were washed using PBS and incubated with 2-NBDG for 15 min followed by washing with cold PBS for 2–3 times. The images were then viewed and captured using Olympus fluorescence microscope. For quantification, the 2-NBDG-treated cells were lysed by incubating with 60 μL passive lysis buffer (Promega Corporation, Madison, WI, USA), for 10 min. The cell lysates were then transferred into 96-well black plate and fluorescence RFU was read at 465/540 nm using EnVision microplate reader, Perkin Elmer (Boston, MA, USA).

### 4.9. GLUT4 Translocation Assay

C2C12 myotubes were cultured in 8-well chamber slides, differentiated and cells were serum starved overnight. Then, cells were treated with either 1 µM or 2 µM THCB compound or 0.5% (*v*/*v*) DMSO for 24 h followed by washing once using KRPH buffer and fixed in 4% (*w*/*v*) paraformaldehyde for 1 h. Subsequently, the cells were washed with 150 μL MAXwash^TM^ washing medium (Active Motif, Carlsbad, CA, USA), and background signals were blocked using 100 μL MAXblock^TM^ blocking medium (Active Motif, Carlsbad, CA, USA) for 30 min at 37 °C. Cells were then washed again three times using 150 μL MAXwash^TM^ washing medium. Subsequently, 1 μL GLUT4 antibody diluted in 100 μL MAXstain^TM^ staining medium (Active Motif, Carlsbad, CA, USA) was transferred to the cells and incubated overnight. Cells were then washed using MAXwash^TM^ washing medium and incubated with 1 μL secondary antibody diluted in 200 μL MAXstain^TM^ staining solution for 1 h. Cells were then rinsed three times using MAX wash^TM^ washing solution. Finally, cells were mounted in the Fluoroshield with DAPI, covered with a glasscover slip and viewed under the confocal microscope.

### 4.10. Statistical Analysis

One way ANOVA was used to calculate the significant differences (*p* < 0.05) for mammalian cell-based PPARγ ligand screening assay, whereas paired *t*-test was used to calculate the significant difference for gene expression analysis. Data are reported as mean ± SEM. All statistical analysis were conducted using Minitab 15 statistical software (version 15.1.1.0, State College, PA, USA).

## 5. Conclusions

In conclusion, THCB, a natural compound isolated from *M. charantia*, was demonstrated to act as PPARγ ligand by its ability to induce PPRE transcriptional activity, increase the expression of PPARγ target genes and induce 3T3-L1 pre-adipocytes differentiation to adipocytes, albeit at lower levels. Furthermore, this natural compound increased glucose uptake by increasing the translocation of Glut4 to cell surface probably via insulin-dependent or independent pathway. Therefore, THCB may offer a potential to be developed as a therapeutic agent against type II diabetes with less side effects.

## Figures and Tables

**Table 1:** Comparison of the amino acid residues arising due to hydrogen bond and Van de waals interaction at the binding site of PPARγ with the presence of rosiglitazone and THCB.

Type of Interaction	Ligand	Amino Acid Residue that Contribute to Binding of Ligand Towards PPARγ
Hydrogen Bond	Rosiglitazone	SER342, SER289, CYS 285
	THCB	ARG280, GLN283
Van der waals	Rosiglitazone	GLY284, TRY327, PHE363, GLN286, LEU453, LEU469, LEU465, TYR473, HIS323, ILE326, VAL339, ILE341
	THCB	GLU259, ILE281, LEU255, GLN273, GLY284, LYS275, HIS 466

## Data Availability

The data presented in this study are available in the article.

## Sample Availability

Samples of the compounds are not available from the authors.
